# A Novel and Easy-to-Promote Prognostic Model for Patients With Uveal Melanoma

**DOI:** 10.3389/fonc.2022.879394

**Published:** 2022-06-02

**Authors:** Han Yue, Binbin Xu, Jian Gao, Yingwen Bi, Kang Xue, Jie Guo, Rui Zhang, Hui Ren, Yifei Yuan, Jiang Qian

**Affiliations:** ^1^Department of Ophthalmology, Eye & Ear, Nose, and Throat (ENT) Hospital of Fudan University, Shanghai, China; ^2^Shanghai Key Laboratory of Visual Impairment and Restoration, Fudan University, Shanghai, China; ^3^Center of Clinical Epidemiology and Evidence-based Medicine, Fudan University, Shanghai, China; ^4^Department of Pathology, Eye & Ear, Nose, and Throat (ENT) Hospital of Fudan University, Shanghai, China

**Keywords:** uveal melanoma (UM), prognostic model, nomogram, survival probabilities, external validation

## Abstract

**Purpose:**

To establish an easy and widely applicable prognostic prediction model for uveal melanoma (UM) based on a Chinese population.

**Patients and Methods:**

A total of 295 consecutive cases treated at the Eye & ENT Hospital of Fudan University were included as the primary cohort, and 256 cases were included in the validation cohorts from two external Caucasian databases. Clinicopathological data were collected retrospectively, and nomogram models were formulated based on multivariable analysis. The concordance index (C-index), AUC (area under the Receiver Operating Characteristic, ROC curve), and Brier score were calculated and compared.

**Results:**

Based on the training cohort, a nomogram model was established with five relevant variables: age, tumor size, ciliary body involvement, non-spindle cell type and extra-scleral extension. The C-index was 0.737, the 3- and 5-year AUCs were 0.767 and 0.742, and the Brier scores for 3- and 5-year survival were 0.082 and 0.129, respectively, which showed superior prediction compared to that of the Tumor, Node and Metastasis staging system. The model also displayed good discrimination and calibration in the external validation cohorts. By risk stratification, patients could be divided into low- and high-risk groups, and the overall survival curves displayed significant differences in the training and validation cohorts.

**Conclusion:**

Our nomogram model was simple and accurate at predicting the overall survival of patients with UM. It was established based on Asian patients and proved suitable for Caucasian patients; thus, it has a wide range of potential applications, especially for patients living in less medically developed countries and regions.

## Introduction

Uveal melanoma (UM) is the most common intraocular primary malignancy in adults involving the choroid, iris and ciliary body, and has a high tendency to metastasize, resulting in high mortality (1–3). Approximately half of the patients develop metastatic disease during a long follow-up (1, 2). Currently, there is no proven effective treatment for patients with metastasis. Once metastasis is detected, the patient survival rate rapidly decreases to approximately 15% at one year, and the median survival time is estimated to be 4 to 15 months (4). Some clinical trials are being conducted with patients with metastasis, which may provide some benefits for patients (5, 6). Therefore, early screening of high-risk patients and early detection of metastasis are very important. Moreover, establishing risk stratification and making different follow-up plans accordingly can also save many medical resources, reduce unnecessary invasive examinations and testing expenses, and thus result in rational allocation of medical resources.

Worse prognostic indicators for UM include 1) clinical factors, such as older age at presentation, male sex, larger basal tumor diameter, high tumor thickness, ciliary body involvement, and extraocular spread (7, 8); 2) histopathological factors, such as epithelioid melanoma cytomorphology, certain extravascular matrix patterns such as closed loops (9), a high mitotic count, and high tumor microvascular density (8); and 3) genetic abnormalities, including chromosomal changes (chromosome 3 loss, 8q gain, 1p loss and 6p gain) (8), class 2 gene expression profiles (10), and other biomarkers (BAP1, SF3B1, EIF1AX, and PRAME) (11, 12). Most of the prognostic prediction models have been established based on these risk indicators, such as the TNM staging system, the gene expression profile (10, 13), the Liverpool Uveal Melanoma Prognosticator Online (14), the Cancer Genome Atlas Classification (15) and other models (16–19). Most of the present prognostic models are relatively complicated and less feasible. The high expenses of these tests may also be limitations for some patients, especially those living in developing regions and countries.

To our knowledge, no prognostic models have been analyzed and established based on Asian patients. Asian patients, compared to the Caucasian population, have some unique features, such as a lower incidence probability, earlier onset age, and larger tumor diameter (20, 21). The two largest clinical prognosis studies in China showed that Chinese patients have a younger onset age and no sex difference and tend to have a better prognosis (22, 23). Studies from other Asian countries (Japan (24), India (25), Korea (26) and Singapore (27) also showed similar results.

Based on the long-term follow-up results of the patients in our hospital, we established a novel and simple nomogram prediction model to predict the survival of UM patients. Two public databases from Western countries were analyzed for external verification.

## Methods

### Patients and Clinicopathological Data of the Training Cohort

We retrospectively and consecutively reviewed all patients with UM who were treated between January 2002 and October 2017 at the Eye & ENT Hospital of Fudan University in Shanghai, one of the three largest tertiary eye centers in China. All patients were diagnosed by experienced ocular surgeons. All patients received enucleation or proton radiotherapy during this time period. Informed consent was obtained from the subjects after explanation of the nature and possible consequences of the study. Institutional Review Board (IRB)/Ethics Committee approval was obtained, and all data for the study were collected and analyzed in accordance with the tenets set forth in the Declaration of Helsinki.

Patients’ clinical data included age at diagnosis, sex, laterality, best corrected visual acuity (BCVA), tumor location, largest basal tumor diameter and thickness determined by type B ultrasonography and survival status. The tumor classification of each patient was recorded according to the seventh edition of the Tumor, Node and Metastasis (TNM) staging system. All tumor specimens stained with hematoxylin and eosin (H&E) were examined by one experienced pathologist to identify the cell type, tumor location and extraocular spread (extra-scleral extension) pathologically. Based on the modifications of Callender’s classification of UM (28), cell types were classified as (1) spindle, epithelioid and mixed; and (2) spindle, and non-spindle types (epithelioid and mixed). In addition, overall survival (OS) time was recorded.

### Data Collection From the Validation Cohort

The external validation data came from The Cancer Genome Atlas (TCGA) database and the Surveillance, Epidemiology, and End Results (SEER) database. From the SEER database, we selected all patients diagnosed with “ciliary body melanoma”, “choroid melanoma” or “iris melanoma”, together with the 7^th^ edition of the TNM classification.

### Statistical Analysis

Quantitative variables are expressed as the means ± standard deviations. Frequency counts and percentages of participants within each category were calculated for categorical data. Kaplan–Meier curves were used to analyze the survival data. Univariate and multivariate Cox proportional hazards regression was performed to assess factors related to patient survival. Hazard ratios (HRs) and their corresponding 95% confidence intervals (CIs) were derived from Cox models. The proportionality assumption of the Cox model was assessed by testing the correlation of the Schoenfeld residuals and the logarithm of time using the cox-zph test in R.

We searched for predictors of OS that were repeatedly reported in studies or systematic reviews and can be easily ascertained in different settings with various levels of clinical experience. As described herein, 14 variables shown in **Table 1** were entered into the selection process. We used a backward stepwise elimination approach with a total of five elimination steps to simplify the model on the basis of the Akaike information criterion. The criterion estimates the fit of each statistical model, penalizes overfitting, and provides a means to select relevant variables that improve the model even if they do not reach the threshold for significance (P<0.05) (29). The continuous variables in the primary regression model were tested for linearity. The global test of any interaction was not statistically significant, so the full prediction model included only main effects. On the basis of the results of the multivariable analysis, a nomogram was formulated by the R package.

**Table 1:** Demographics and clinicopathologic characteristics of the patients in the training cohort and the validation cohorts.

Variables	Training cohort	Validation cohorts		P value
	China	SEER	TCGA	
	(n = 295)	(n = 179)	(n = 77)	
**Age (years),** yrs.	49.4 ± 13.8	61.2 ± 14.1	61.7 ± 14.1	<0.001
**Largest basal diameter,** mm	12.6 ± 3.6			
**Thickness,** mm	8.8 ± 3.1			
**Sex,** no. (%)				0.010
Female	157 (53.2)	70 (39.1)	34 (44.2)	
Male	138 (46.8)	109 (60.9)	43 (55.8)	
**Laterality,** no. (%)				0.635
Right	136 (46.1)	87 (48.6)		
Left	159 (53.9)	92 (51.4)		
**BCVA,** no. (%)				
≤0.05	147 (49.8)			
>0.05, ≤0.3	81 (27.5)			
>0.3	76 (22.7)			
**Ciliary body involvement,** no. (%)				<0.001
No	250 (84.8)	119 (66.5)	59 (76.6)	
Yes	45 (15.2)	60 (33.5)	18 (23.4)	
**Iris involvement,** no. (%)				0.0157
No	288 (97.6)	173 (96.7)		
Yes	7 (2.4)	6 (3.3)		
**Cell types,** no. (%)				0.002
Spindle	169 (57.3)		28 (36.4)	
Epithelioid	40 (13.6)		12 (15.6)	
Mix	86 (29.1)		37 (48.0)	
**Non-spindle cell type,** no. (%)				0.005
Spindle	169 (57.3)	96 (53.6)	28 (36.4)	
Non-spindle	126 (42.7)	83 (46.4)	49 (63.6)	
**Extra-scleral extension,** no. (%)				0.020
No	282 (95.6)	159 (88.8)	71 (92.2)	
Yes	13 (4.4)	20 (11.2)	6 (7.8)	
**Tumor size categories,** no. (%)				<0.001
T1	16 (5.4)	28 (15.6)	0 (0.0)	
T2	104 (35.3)	49 (27.4)	5 (6.5)	
T3	129 (43.7)	61 (34.1)	34 (44.2)	
T4	46 (15.6)	41 (22.9)	38 (49.3)	
**TNM stages,** no. (%)				<0.001
I	13 (4.4)	15 (8.4)	0 (0.0)	
IIA	91 (30.8)	49 (27.4)	4 (5.2)	
IIB	120 (40.7)	48 (26.8)	29 (37.7)	
IIIA	51 (17.3)	40 (22.3)	29 (37.7)	
IIIB	20 (6.8)	20 (11.2)	11 (14.3)	
IIIC	0 (0.0)	6 (3.3)	1 (1.3)	
IV	0 (0.0)	1 (0.6)	3 (3.9)	
Extent of the disease^*^, no. (%)				<0.001
A	239 (81.0)	105 (58.7)	56 (72.7)	
B	43 (14.6)	54 (30.2)	15 (19.5)	
C	11 (3.7)	11 (6.1)	3 (3.8)	
D	2 (0.6)	6 (3.4)	2 (2.6)	
E	0 (0.0)	3 (1.7)	1 (1.3)	

^*^Extent of the disease: A: without ciliary body involvement and extraocular extension; B: with ciliary body involvement; C: without ciliary body involvement but with extraocular extension ≤ 5 mm in diameter; D: with ciliary body involvement and extraocular extension ≤ 5 mm in diameter; E: Any tumor size category with extraocular extension > 5 mm in diameter.

We assessed the predictive accuracy of the prognostic nomogram in the internal testing set and two external validation cohorts with discrimination and calibration. Discrimination for predicting outcome was evaluated by calculating the Harrell concordance index (C-index) (30), together with the area under the time-dependent ROC curve (AUC). The 95% CIs were generated with bootstrapping methods to account for residual uncertainty. Calibration of the nomogram for 3-, 5-, and 10-year OS was assessed with calibration plots and Brier scores. A 95% perfect calibration is implied by a 45° diagonal line, whereas relevant deviation above or below this line reflects underprediction or overprediction. We determined the clinical significance of using nomograms by performing decision curve analysis (DCA) (31).

We did not impute missing information because there were no missing values in the derivation cohort. To classify individuals into high-risk and low-risk categories, individual predicted risks were converted into binary categories using a cutoff value. The optimal cutoff value was determined using the maximally selected rank statistics from the “survminer” R package. Decision curve analysis was performed using the “rmda” R package. The significance level was set at P<0.05 (two-sided probability). All analyses were performed using R version 4.0.1 (http://www.rproject.org). The results are reported in compliance with the Transparent Reporting of a multivariable prediction model for Individual Prognosis or Diagnosis (TRIPOD) criteria (**Supplementary Table S1**).

## Results

### Screening Process and Clinicopathologic Characteristics of Patients

In total, 318 patients were treated in our hospital in this period. All the patients were Asian. Patients without complete follow-up data (4.4%) and patients treated by proton radiotherapy lacking pathological data (2.8%) were excluded. Thus, 295 patients, all treated by enucleation, with a mean follow-up time of 70.4 ± 47.5 months (range, 1.5 to 207.7 months) were included in the training cohort. The patients included and excluded were comparable (**Supplementary Table S2**).

**Figure 1:**
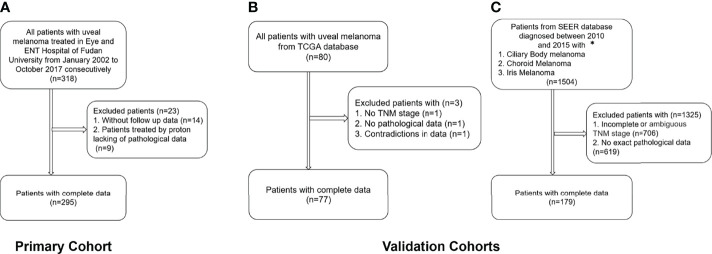
Flow chart of the inclusion and exclusion criteria for the training and validation cohorts. **(A)** Training cohort; **(B)** TCGA validation cohort; **(C)** SEER validation cohort. *Patients with the 7th edition of the TNM classification were diagnosed between 2010 and 2015 in the SEER database.

In the TCGA database, excluding three patients without TNM staging or pathological data or with contradictory data, 77 patients with a mean follow-up time of 26.2 ± 17.5 months (range, 0.1 to 86.7 months) were included. In the SEER database, patients with incomplete or ambiguous TNM stage or patients without exact pathological data were excluded, and a total of 179 patients with a mean follow-up time of 44.0 ± 21.6 months (range, 1 to 94 months) were analyzed (**Figure 1**).

**Figure 2:**
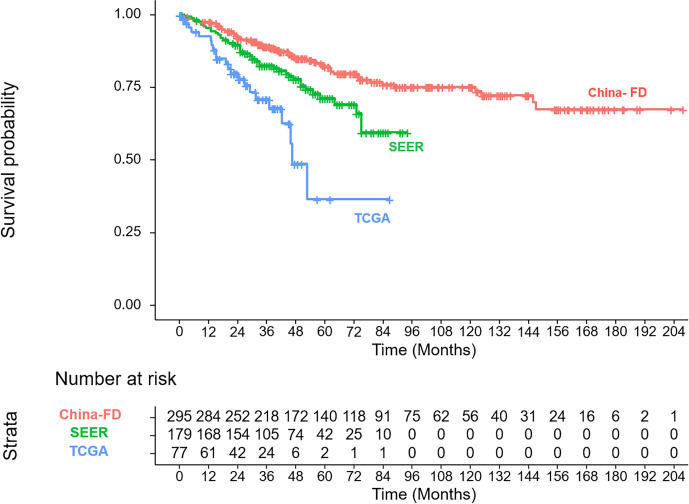
The overall survival curves for the training and validation cohorts.

The clinicopathologic characteristics of the patients in the training cohort and the validation cohorts are listed and compared in **Table 1**. In the three cohorts, our patients showed a younger onset age, lower male tendencies, less non-spindle cell type, less ciliary body involvement and less extra-scleral extension. The corresponding OS curves are shown in **Figure 2**.

### Independent Prognostic Factors in the Training Cohort

By Cox multivariate regression analysis in the training cohort, among all the clinicopathologic variables, the five most relevant variables were screened out as age, largest basal diameter, ciliary body involvement, non-spindle cell type and extra-scleral extension, and accordingly, prediction Model I was established (**Table 2**).

**Table 2:** Cox multivariate regression analysis of the prediction model in the training cohort.

Variables	Model I			P value	Model II			
	β	Se	HR (95% CI)		β	Se	HR (95% CI)	P value
Age	0.018	0.009	1.02 (1.00,1.04)	0.053	0.017	0.009	1.02 (1.00,1.04)	0.075
Largest basal diameter	0.109	0.036	1.15 (1.04,1.20)	0.002				
Ciliary body involvement	0.699	0.320	2.01 (1.07,3.77)	0.029	0.676	0.333	1.97 (1.02,3.78)	0.042
Non-spindle cell type	0.806	0.286	2.24 (1.28,3.92)	0.005	0.888	0.283	2.43 (1.40,4.23)	0.002
Extra-scleral extension	1.453	0.421	4.28 (1.87,9.76)	<0.001	1.693	0.437	5.44 (2.31,12.80)	<0.001
Tumor size categories								
1					–	–	1.00	–
2					0.329	0.759	1.39 (0.31,6.14)	0.664
3					0.842	0.743	2.32 (0.54,9.95)	0.257
4					1.343	0.756	3.83 (0.87,16.87)	0.076

In the TNM staging system, the largest basal diameter and tumor height were combined as the tumor size. In the TCGA and SEER databases, tumor size was recorded. Thus, to facilitate external validation and maintain consistency, we developed Model II in which tumor size was included instead of the largest basal diameter. Although the tumor size categories did not reach the threshold for significance (P<0.05), the HR markedly increased with tumor size, indicating that it could also serve as a predictor in the prediction model (**Table 2**, Model II). We compared the discrimination and calibration between Model II and Model I and compared them with the traditional TNM staging system.

### Prognostic Nomogram for OS

**Figure 3:**
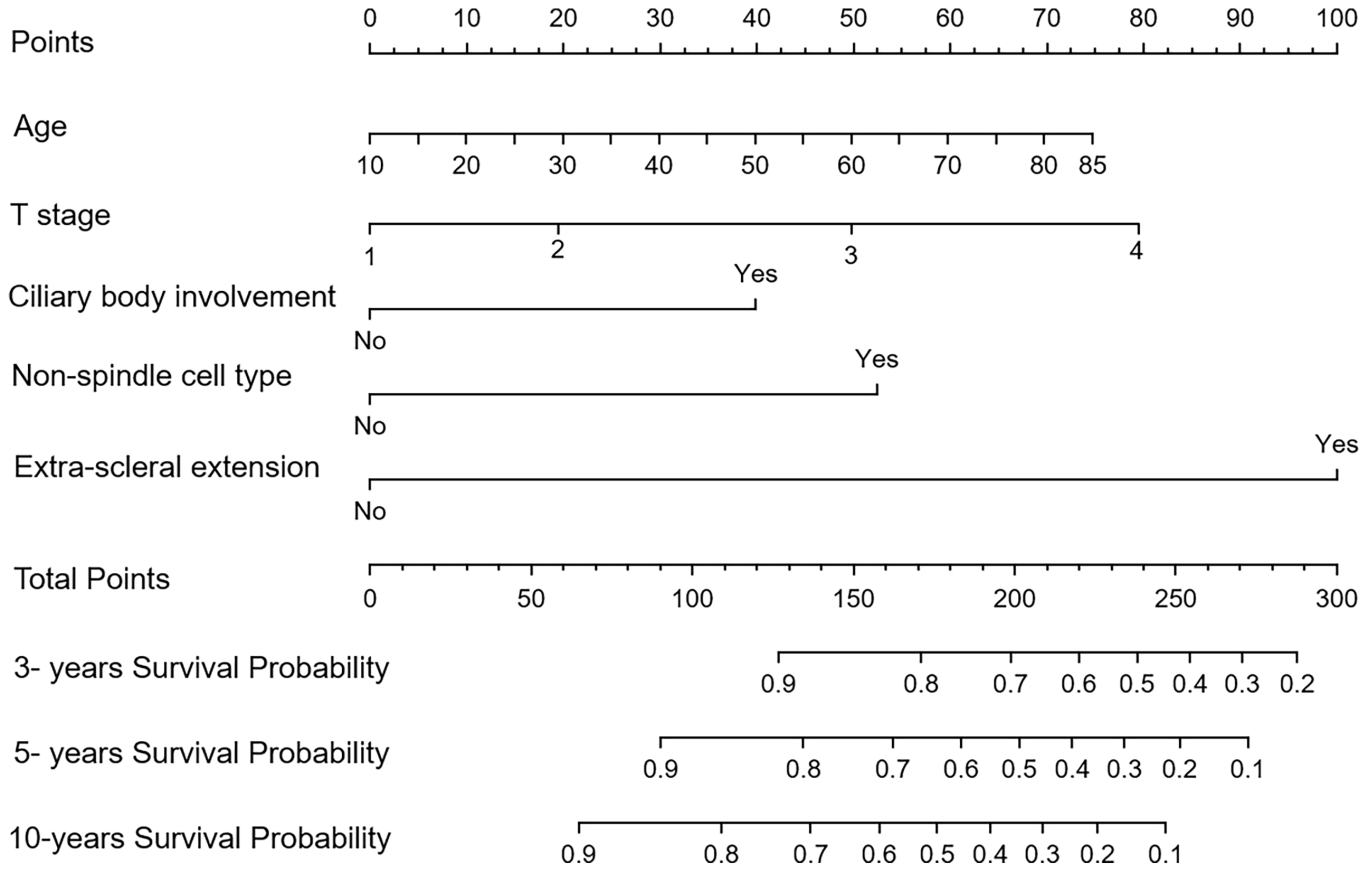
The nomogram for the overall survival of patients in the training cohort (Model II).

The nomograms for Model I and Model II incorporating the prognostic factors were established. In the nomograms, each subtype within the variable was assigned a score on the point scale. Summing up the scores and then locating them on the total points scale, we could easily draw a straight line down to the estimated probability of survival at each time point for each patient (**Figure 3**).

### Discrimination, Calibration and Validation of the Nomogram Model

**Figure 4:**
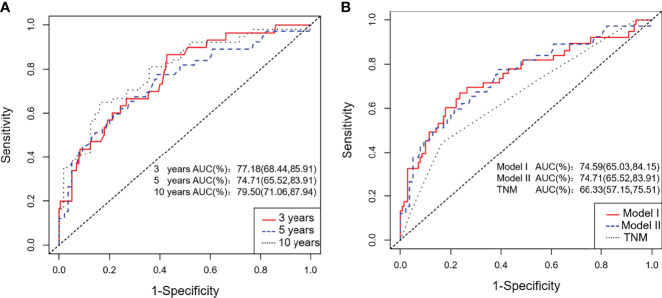
ROC curves for the training cohort. **(A)** The 3-, 5-, and 10-year ROC curves of the training cohort; **(B)** ROC curves of Nomogram Model I, Model II and the TNM staging system for the training cohort.

We calculated two validation measures of accuracy: discrimination and calibration. In the training cohort, the C-index and 3- and 5-year AUC for the established Model I and Model II were significantly higher than those of the TNM staging system (**Table 3**, **Figure 4**). For the calibration, Models I and II showed the same good results, and both were better than the TNM staging system (**Table 3**).

**Table 3:** Internal and external validations of the prediction models.

Discrimination		TNM stage	Model I	Model II
C-index (95% CI)	Training cohort	0.652 (0.580,0.725)	0.730 (0.660,0.800)	0.737 (0.672,0.801)
	Internal Validation (Bootstrap)	0.643 (0.570,0.716)	0.726 (0.657,0.795)	0.730 (0.661,0.793)
	External Validation: TCGA	0.632 (0.480,0.783)	NA	0.747 (0.622,0.872)
	External Validation: SEER	0.705 (0.615,0.794)	NA	0.798 (0.721,0.875)
3-year AUC (95% CI)	Training cohort	0.681 (0.580,0.782)	0.763 (0.670,0.857)	0.772 (0.684,0.859)
	Internal Validation (Bootstrap)	0.672 (0.556,0.737)	0.756 (0.664,0.850)	0.767 (0.679,0.853)
	External Validation: TCGA	0.615 (0.444,0.787)	NA	0.800 (0.669,0.931)
	External Validation: SEER	0.682 (0.574,0.790)	NA	0.795 (0.706,0.884)
5-year AUC (95% CI)	Training cohort	0.663 (0.572,0.755)	0.746 (0.650,0.841)	0.747 (0.655,0.839)
	Internal Validation (Bootstrap)	0.655 (0.566,0.747)	0.741 (0.645,0.835)	0.742 (0.650,0.833)
	External Validation: TCGA	0.700 (0.409,0.961)	NA	0.770 (0.475,0.941)
	External Validation: SEER	0.784 (0.694,0.882)	NA	0.891 (0.822,0.961)
**Calibration**				
3-year Brier score (95% CI)	Training cohort	0.087 (0.055,0.152)	0.079 (0.049,0142)	0.082 (0.053,0.111)
	Internal Validation (Bootstrap)	0.090 (0.070,0.161)	0.082 (0.054,0150)	0.095 (0.064,0.158)
	External Validation: TCGA	0.175 (0.118,0.311)	NA	0.114 (0.097,0.168)
	External Validation: SEER	0.126 (0.071,0.161)	NA	0.092 (0.062,0.121)
5-year Brier score (95% CI)	Training cohort	0.121 (0.085,0.174)	0.119 (0.086,0152)	0.123 (0.090,0.157)
	Internal Validation (Bootstrap)	0.125 (0.084,0.180)	0.120 (0.087,0154)	0.129 (0.092,0.167)
	External Validation: TCGA	0.193 (0.127,0.355)	NA	0.167 (0.110,0.189)
	External Validation: SEER	0.137 (0.077,0.169)	NA	0.104 (0.075,0.149)

NA, not available.

**Figure 5:**
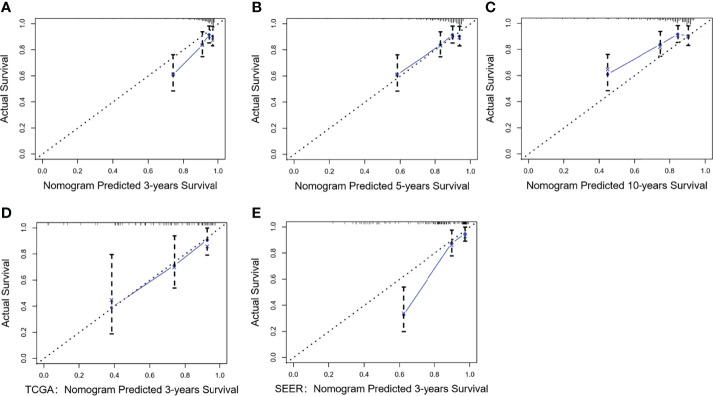
Calibration curves of Model II for the training and validation cohorts. Nomogram-predicted OS is plotted on the x-axis; actual OS is plotted on the y-axis. A plot along the 45° line indicates a perfect calibration model in which the predicted OS is identical to the actual outcomes. **(A–C)** The 3-, 5-, and 10-year calibration curves for the training cohort; **(D)** The 3-year calibration curve for the TCGA validation cohort; **(E)** The 3-year calibration curve for the SEER validation cohort.

Considering that Model I and Model II were comparable in the training cohort and that Model II was more suitable for external verification, we chose Model II to conduct subsequent analyses. In the validation cohorts, Model II showed a higher C-index and 3- and 5-year AUCs than the TNM staging system (**Table 3**). Its 3- and 5-year Brier scores were lower than those of the TNM staging system, indicating a better calibration of Model II (**Table 3**). The calibration plots of prediction closely approximated the 45° line, which presented good consistency in the primary cohort for 3-, 5-, and 10-year OS and excellent agreement in the validation cohorts between the nomogram prediction and actual observation for 3-year OS (**Figure 5**). Thus, we chose Model II as the final prediction model.

**Figure 6:**
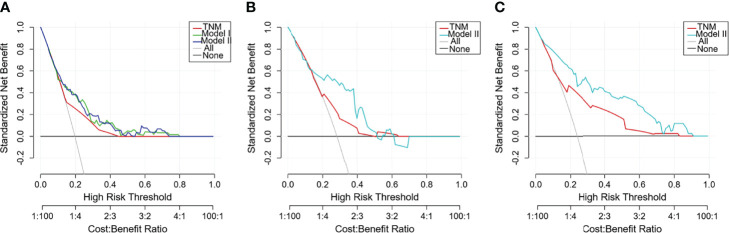
Decision curve analysis (DCA) curves with net benefit score on the vertical axis and high risk thresholds on the horizontal axis for the training and validation cohorts. The net benefit is determined by calculating the difference between the expected benefit and the expected harm associated with each prediction model. The gray line denotes the assumption that all patients had outcome events (death) during follow-up. The dark black line represents the assumption that no patients had outcome events (death) during follow-up. Other curves represent different prediction models. The curve with the highest benefit score at that threshold is the best choice (17). **(A)** Training cohort; **(B)** TCGA validation cohort; **(C)** SEER validation cohort.

DCA was adopted to assess the nomogram’s clinical significance. This method offers insight into clinical consequences on the basis of threshold probability, from which the net benefit could be derived (32). The lines of Model II in **Figure 6** were all far from both extreme curves and from the TNM line in the training cohort and the validation cohorts, which suggested that the Model II nomogram had superior prediction compared to that of the TNM staging system.

### Performance of the Nomogram in Stratifying Patient Risk

**Figure 7:**
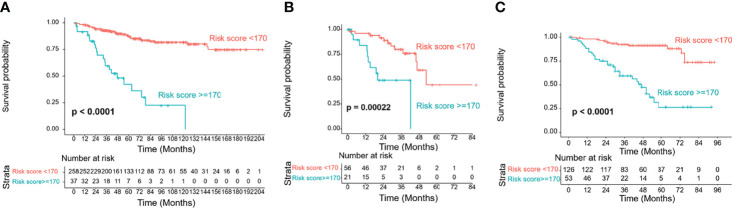
Risk group stratification of the training and validation cohorts by the cutoff value of 170. **(A)** Training cohort; **(B)** TCGA validation cohort; **(C)** SEER validation cohort.

Based on the maximally selected rank statistics in the training cohort, the optimal cutoff value was determined to be 170. Accordingly, our patients could be divided into two groups: low-risk and high-risk groups. Their OS curves displayed the most significant difference, with a P value <0.0001 (**Figure 7A**). In the validation cohorts, risk stratification also showed a significant distinction between the two groups (**Figures 7B, C**).

## Discussion

The TNM staging system is used worldwide for UM and integrates the basal tumor diameter, tumor height, tumor location, extraocular extension, lymph glands and distant metastasis and could thus be used as a prediction model. The Cancer Genome Atlas Class groups tumors as A, B, C or D based on their chromosome status and can also be used to predict metastasis and death (15, 33). The class 1 and class 2 gene expression profiles are genetic assessment tools that are widely applied in Western countries, especially in the United States. Based on genetic analysis, the gene expression profile can distinguish patient prognosis with a significant difference in that nearly all metastatic deaths fall into the class 2 category, and it has been validated on multiple independent datasets (13, 34). Another promising estimating model is the Liverpool Uveal Melanoma Prognosticator Online, which combines pathological, clinical and genetic data to provide individual patients with an estimated survival time as well as the risk of metastasis (14). This model has been validated with several international cohorts and can serve as a valuable tool for predicting all-cause mortality (35, 36). In addition, there are several other prognostic models, such as the artificial neural network by Kaiserman (16), the nomogram-SEER model by Zeng (17), the parsimonious model by Damato (18), and the Markov multistate model by Eleuteri (19). The main characteristics of these models are listed in **Table 4**. Although gene and chromosome testing increase the accuracy of predictions, the high expense and intricate testing technology may limit their wide application in developing regions and countries. Moreover, from **Table 4**, we found that these models were developed mostly with the Caucasian population.

**Table 4:** Prognostic prediction models for uveal melanoma.

Prognosis models	Variables	Data resource/n	Access method	Main outcome	Risk stratification	Validation
TNM staging system	Tumor location, LBTD, thickness, extraocular extension, lymph glands, distant metastasis	American Joint Committee on Cancer	Staging Form	Stage and Grouping	I, IIA, IIB, IIIA, IIIB, IIIC, IV	Multiple centers
Gene expression profile (13, 34)	Gene expression profile; expression of 15 genes	NA/25; 3 genechip platforms/65	Commercial: DecisionDx-UM test	Class 1A, Class 1B, Class 2	Class 1A, Class 1B, Class 2	Multiple centers
The Cancer Genome Atlas Classification (15, 33)	Chr3, Chr8	TCGA database/80	TCGA Class Table	Class A, B, C, D	Class A, B, C, D	Multiple centers
LUMPO (LUMPO3) (14)	Clinical: age, sex, LBTD, thickness, anterior margin, extraocular extension Pathological: cell type, closed loops, mitotic count Genetic: Chr3, Chr8	Mainland Britain/3658	Online	All-cause mortality	No	Multiple centers
Artificial neural network (16)	Demographics, LBTD, thickness, internal reflectivity by ultrasonography, regularity, vascularity, extra-scleral extension, liver function, liver imaging	Israel/153		5-year mortality	No	No
Nomogram-SEER (17)	Age, histological type, T stage, M stage	SEER database/588 (training cohort) and 251 (validation cohort)	Nomogram model table	1-, 3-, and 5-year cause-specific survival	Low-risk, high-risk	Internal validation, no external validation
Parsimonious model (18)	LBTD, Chr3	England, Scotland, and Wales/8348	Risk Table	2-, 5-, and 10-year metastatic mortality	No	No
Markov multistate model (19)	Age, sex, anterior margin position, LBTD, thickness, extraocular extension, cell type, closed loops, mitotic count, Chr3, Chr8	England, Scotland and Wales/4161		2- and 5-year metastatic death survival	No	No

LBTD, largest basal tumor diameter; Chr3, chromosome 3 status; Chr8, chromosome 8 status; NA, not available.

Our prediction models screened out only five clinicopathologic variables and did not include gene testing or chromosome typing. However, this model still achieves rather high prediction accuracy and showed even better discrimination and calibration than those of the TNM staging system in our patients. This result suggested that these clinicopathologic features, even without the genetic phenotypes, highly represent the inherent characteristics of the disease, further indicating that the gene phenotypes may have been outwardly expressed by these clinicopathologic features in our patients. Thus, the prediction accuracy was not sacrificed when evaluating without genetic testing. Our model was very easy to operate and has low requirements for testing equipment and a low testing cost; hence, it is highly feasible for use by patients from less medically developed countries and regions. Moreover, we conducted validation in the TCGA and SEER databases, and the model showed good discrimination and calibration in these two databases, which indicated that our nomogram model is also suitable for Western patients.

Comparing the baseline of our patients with the Western databases, the clinicopathology and prognosis of our patients were different: our patients had a younger onset age, less male tendencies, less non-spindle cell type, less ciliary body involvement and less extra-scleral extension, and our patients showed a better survival trend. It is inferred that the better prognosis may be related to the inherent genetic features. A study sequenced the whole genome of Chinese patients and found that the GNA11 mutation rate was only 21.5% in those patients, which was much lower than the reported rate of 32.6% in a Caucasian population; instead of BAP1, SF3B1, and EIF1AX, their patients with HIF1A and FOXO1 mutations exhibited worse OS (37). This finding suggests that there may be inherent genetic differences between the two groups of patients.

Based on this model, the risk stratification displayed an obvious distinction between the low- and high-risk groups in both the training and validation cohorts. This result suggests that the model could provide an excellent differential ability in prognosis prediction. Thus, high-risk patients could be easily screened out and closely followed up to detect and treat metastasis earlier. Furthermore, we found that the curves in the training cohort were very flat and smooth, especially the one indicating the low-risk group, while the corresponding curves in the TCGA and SEER cohorts showed a decreasing tendency. This result indicated that this model reflected the prognosis of Chinese patients well, while there may be other independent variables affecting the prognosis of Caucasian patients. Therefore, it may also serve as proof that there are physiological differences between Asian and Caucasian patients.

There are some limitations of this study: 1. Although we used two external databases, the follow-up time of these two databases was limited, so we can only verify the 5-year survival rate instead of the long-term prediction effect of the 10-year survival rate. 2. We only selected patients with complete information, especially in the SEER database, which could have potentially introduced selection bias. 3. Due to the characteristics of the retrospective study, we didn’t compare the efficiency of our model with other UM prediction models. In the future, some prospective studies will be carried out to further verify the efficacy of the present model, and its comparisons with other UM prediction models will be designed as well.

Model II has the following strengths: 1. This model was established based on Asian patients with UM, and also proved applicable to the Caucasian population, thus indicating its wide scope of application. 2. The model requires only a few variables, which are easy to obtain clinically. It could not only provide the estimated probability of survival for each patient, but also differentiate the low- and high-risk patients (with <170 or ≥170 cores) and thus make different follow-up plans accordingly. For example, we could reduce unnecessary invasive examinations and testing expenses for low-risk patients, and give close follow-ups or preventive treatments, such as clinical drug trials, to patients with high risk. In this way, the model is simple to operate and will contribute to rational allocation of medical resources.

In conclusion, we developed a simple and easy-to-promote nomogram model to predict the OS of patients with UM based on our Chinese population, and it was more accurate than the TNM staging system. Our prediction model also showed high discrimination and good calibration when verified by two Caucasian databases, suggesting that the model is suitable for a wide population.

## Data Availability Statement

The raw data supporting the conclusions of this article will be made available by the authors, without undue reservation.

## Ethics Statement

The studies involving human participants were reviewed and approved by Ethics Committee of Eye & ENT Hospital of Fudan University. The patients/participants provided their written informed consent to participate in this study.

## Author Contributions

Conception and design (HY and JQ); Data collection (HY, BX, YB, KX, JGu., RZ, HR, YY, and JQ); Data analysis and interpretation (JGa.); Manuscript preparation (HY, BX, and JQ); Obtained funding (KX). All authors contributed to the article and approved the submitted version.

## Funding

Project supported by the Shanghai Committee of Science and Technology, China (Grand No. 20Y11911200). The funding had no role in the design or conduct of this research.

## Conflict of Interest

The authors declare that the research was conducted in the absence of any commercial or financial relationships that could be construed as a potential conflict of interest.

## Publisher’s Note

All claims expressed in this article are solely those of the authors and do not necessarily represent those of their affiliated organizations, or those of the publisher, the editors and the reviewers. Any product that may be evaluated in this article, or claim that may be made by its manufacturer, is not guaranteed or endorsed by the publisher.
