# Supplementary material for: A Novel and Easy-to-Promote Prognostic Model for Patients With Uveal Melanoma
Source: Front Oncol. 2022 Jun 2;12:879394. doi: 10.3389/fonc.2022.879394 (PMC9201029; doi:10.3389/fonc.2022.879394)
Supplement: Supplementary file 2 [file Table_2.docx]

Supplementary Table S2: Comparison of the basic characteristics between patients included and excluded in our consecutive case series in this study.

| **Variables** | **Patients included** | **Patients excluded** | **P value** |
| --- | --- | --- | --- |
|  | (n=295) | (n=23) |  |
| **Age (years),** yrs | 49.4±13.8 | 47.5±11.9 | 0.521 |
| **Largest basal diameter,** mm | 12.6±3.6 | 12.8±3.9 | 0.724 |
| **Thickness,** mm | 8.8±3.1 | 7.8±2.9 | 0.137 |
| **Sex,** no.(%) |  |  | 0.923 |
| Female | 157(53.2) | 12(52.2) |  |
| Male | 138(46.8) | 11(47.8) |  |
| **Laterality,** no.(%) |  |  | 0.518 |
| Right | 136(46.1) | 9(39.1) |  |
| Left | 159(53.9) | 14(60.9) |  |
| **BCVA,** no.(%) |  |  |  |
| ≤0.05 | 147(49.8) | 9(39.1) | 0.262 |
| >0.05, ≤0.3 | 81(27.5) | 10(43.5) |  |
| >0.3 | 76(22.7) | 4(17.4) |  |
| **Ciliary body involvement,** no.(%) |  |  | 0.785 |
| No | 250(84.8) | 19(82.6) |  |
| Yes | 45(15.2) | 4(17.4) |  |
| **Iris involvement,** no.(%) |  |  | 0.560 |
| No | 288(97.6) | 22(95.7) |  |
| Yes | 7(2.4) | 1(4.3) |  |
| **Cell types,** no.(%) |  |  |  |
| Spindle | 169(57.3) | 8(57.1) | 0.993 |
| Epithelioid | 40(13.6) | 2(14.3) |  |
| Mix | 86(29.1) | 4(28.6) |  |
| **Non-spindle cell type,** no.(%) |  |  | 0.991 |
| Spindle | 169(57.3) | 8(57.1) |  |
| Non-spindle | 126(42.7) | 6(42.9) |  |
| **Extra-scleral extension,** no.(%) |  |  | 0.422 |
| No | 282(95.6) | 14(100.0) |  |
| Yes | 13(4.4) | 0(0.0) |  |
| **Tumor size categories,** no.(%) |  |  | 0.396 |
| T1 | 16(5.4) | 3(13.1) |  |
| T2 | 104(35.3) | 7(30.4) |  |
| T3 | 129(43.7) | 11(47.8) |  |
| T4 | 46(15.6) | 2(8.7) |  |
| **TNM stages,** no.(%) |  |  | 0.571 |
| I | 13(4.4) | 2(8.7) |  |
| IIA | 91(30.8) | 8(34.8) |  |
| IIB | 120(40.7) | 8(34.8) |  |
| IIIA | 51(17.3) | 5(21.7) |  |
| IIIB | 20(6.8) | 0(0.0) |  |
| **Extent of the disease *,** no.(%) |  |  | 0.769 |
| A | 239(81.0) | 19(82.6) |  |
| B | 43(14.6) | 4(17.4) |  |
| C | 11(3.7) | 0(0.0) |  |
| D | 2(0.6) | 0(0.0) |  |

*: Extent of the disease: A: without ciliary body involvement and extraocular extension; B: with ciliary body involvement; C: without ciliary body involvement but with extraocular extension ≤ 5 mm in diameter; D: with ciliary body involvement and extraocular extension ≤ 5 mm in diameter.
